# High Precision Human Skin Temperature Fluctuations Measuring Instrument †

**DOI:** 10.3390/s21124101

**Published:** 2021-06-15

**Authors:** Nikolai B. Suvorov, Alexander V. Belov, Konstantin G. Kuliabin, Aleksei A. Anisimov, Timofei V. Sergeev, Oleg A. Markelov

**Affiliations:** 1 Department of Ecological Physiology, Federal State Budgetary Scientific Institution “Institute of Experimental Medicine”, 12 Acad. Pavlov Str., 197376 Saint Petersburg, Russia; nbsuvorov@yandex.ru (N.B.S.); avbelov1@yandex.ru (A.V.B.); brainthrough@gmail.com (K.G.K.); aaanisimov@etu.ru (A.A.A.); sergeev.tv@iemspb.ru (T.V.S.); 2 Department of Biomedical Engineering, Saint Petersburg Electrotechnical University “LETI”, 5 Prof. Popov Str., 197376 Saint Petersburg, Russia

**Keywords:** skin temperature, fluctuations, measuring instrument, pulse wave, electronic circuitry

## Abstract

This paper describes the experimental results of testing a prototype of a high precision human skin rapid temperature fluctuations measuring instrument. Based on the author’s work, an original circuit solution on a miniature semiconductor diode sensor has been designed. The proposed circuitry provides operation in the full voltage range with automatic setting and holding the operating point, as well as the necessary slope of the conversion coefficient (up to 2300 mV/°C), which makes it possible to register fast temperature oscillations from the surface of the human body and other biological objects. Simulation results in the Microcap 12 software and laboratory tests have confirmed all declared design specifications: temperature resolution of 0.01 °C, transducer thermal time constant of 0.05 s. An original thermostat and an experimental setup for the simultaneous registration of the electrocardiogram, pulse wave signals from the Biopac polygraph MP36 and a signal of temperature oscillations from the prototype thermometer have been designed for further investigations. The preliminary test results indicates that using the designed measuring instrument gives a possibility to provide an in-depth study of the relationship between micro- and macro-blood circulations manifested in skin temperature fluctuations.

## 1. Introduction

In modern clinical practice, assessment of the state of blood microcirculation (movement of blood in capillaries and adjacent micro vessels) and evaluation of microcirculatory disorders in the diagnosis of a variety of diseases are relevant [1,2].

This is especially important for diagnostics of the cardiovascular system diseases, in sports and aerospace medicine. For example, due to impaired vascular endothelial function as consequence of atherosclerosis [3], diabetes, cancer, renal disfunction, vasospastic (Raynaud’s syndrome, vibration disease) and other pathologies, including those associated with inflammatory conditions [4]. The diagnosis of such diseases at the initial stages when the changes are still reversible is especially important. The microcirculatory bed is a part of the vascular system, in which these diseases are manifesting in the initial stages.

It remains an unresolved problem for fundamental medicine, to determine the relationship between the blood supply processes at the micro- and macrolevels, as well as the metabolic, thermoregulatory, and other processes that accompany them [5]. Relating to the foregoing, there is a considerable interest in developing and improving tools for reliable and non-invasive monitoring of blood flow in various parts of the body under various conditions [4] and systems capable of recording all vascular elements of the blood stream, i.e., arterial, capillary, and venous components.

Existing methods for recording blood microcirculation can be classified according to the physical principles of measurement: mechanical (plethysmography), optical (photo-plethysmography, laser Doppler flowmetry (LDF) and laser speckle contrast imaging (LSCI)), acoustic ultrasound and thermal (thermometry, thermography). The fundamental feature of microcirculation is its constant variability, which is manifested in spontaneous fluctuations in tissue blood flow. This variability of microcirculation is inherently an objective characteristic of the level of vital activity of tissues. Rhythmic fluctuations in the blood flow and their changes make it possible to obtain information about the specific relationships of various mechanisms that determine the state of microcirculation.

The nature of the oscillatory processes in the microcirculation system is rather complicated. Nowadays it has been established that spontaneous fluctuations in blood flow in tissues are largely, though not only, caused by vasomotions.

The physiological activity causes changes of the body surface temperature in a wide amplitude (60 dB) and frequency range (up to 5 Hz, as shown in Table 1). This activity is associated with heartbeat pulsations which have a ridiculously small amplitude and a maximum frequency of temperature oscillations. Their registration requires high resolution and speed.

The rhythmic structure of flaxemias is the integral result of superposition of various myogenic, neurogenic, respiratory, cardiac, and other indirect effects on the state of microcirculation.

Obviously, information on temperature oscillations associated with microcirculation (movement of blood in capillaries and adjacent microvessels) in all frequency ranges is of great interest for medicine and physiology of visceral systems. Regarding this, it is important to develop methods and tools for studying blood flow oscillations based on the results of skin surface thermometry. At this stage of the investigation, we consider our device as a unique experimental prototype for conducting research in the field of fundamental physiology, not for implementation in clinical practice.

Temperature oscillations (TO) of the human’s body surface contain some information about physiological processes in the frequency range from 0.001 to 2 Hz. This information can assist as a source of data on fluctuations of physiological functions [5,6] (Table 1). Thus, the relations between micro- and macro-blood circulation are manifested in TO. The study of blood circulation is of interest for the physiology of visceral systems and medicine. The solution of scientific and technical problems of registering a TO with high speed and resolution is relevant.

The most significant frequency ranges of signals obtained by three different methods (Heart Rate Variability, Laser Doppler Flowmetry and Temperature Oscillation) are presented in the Table 1 for comparison. Each of these frequency ranges are associated with certain mechanisms of active modulation of blood flow in the microcirculation system. The proximity of these ranges is obvious. The analysis of literature data [7,8] shows that a highly sensitive and fast medical thermometer designed for registration of the fast temperature oscillations of the human body surfaces (skin) should have a resolution of not worse than 0.01 °C in a frequency range of temperature variations of 0.02–5 Hz.

Existing technical solutions do not meet these requirements [9]. Thus, the goal of the work was to develop technical solutions aimed at achieving maximum resolution and high operational properties of a medical thermometer.

Since 1970s thermograms with additional registrations of the heart rate have been reported [10,11,12,13,14]. In these thermometers, a thermocouple was used in the temperature measurement mode, and the heart rate was determined from the electrocardiogram. In the invention of S. Epstein (1985) [15], the temperature measurement was first applied, and, in addition, the temperature change caused by the pulsations of the blood flow is determined. In this method, the temperature of the mucosa of the sublingual region determines the heart rate. However, this technical solution did not find wide practical application due to inconvenience of use.

Semiconductor temperature sensors [16,17,18,19] are effectively used for recording body surface TO. Two types of diode temperature sensors are known. The first of them provides stable direct current of the diode in the static mode, ensuring the steep slope of the conversion voltage to temperature with a temperature coefficient of voltage (TCV) of 2 mV/°C [20,21,22]. Other usage is the pulsed mode [21,22]. During the measurement, the current through the diode changes by a factor of 10 with the measurement of the forward voltage difference from the current change. However, this method has a reduced value of TCV + 0.2 mV/°C.

Thermometers with a resolution of up to 0.001 °C are also known, but they do not have sufficient speed for recording temperature oscillations [23]. One way to increase the speed of the temperature sensor is to preheat the sensor to the expected temperature [24]. Another way is to use two sensors, separated by thermal insulation material and included in the differential scheme [25].

The required thermal time constant of temperature (TCT) is achievable using a semiconductor diode sensor in an open-frame design. The reason for choosing such a sensor is given in the reports of A.V. Belov et al. [10,26,27,28].

This article is an extended version of a thesis given at the FRUCT 2019 conference in Moscow [10]. In that paper, preliminary results of research on the development of a device for recording temperature oscillations, and its potential for application in wearable devices for recording the pulse wave signal have been reported. Since the main focus of the previous work has been on the medical component of the research (within the direction of e-Health and Wellbeing of the mentioned conference), this paper describes in detail its technical component. We moved sequentially, just to make all stages of development as clear as possible: from the basic concept of the original electrical circuit solution, its simulation in Microcap 12, to the actual embodiment in the form of a finished prototype and equipment for laboratory tests (such as the original design of the thermostat).

## 2. Materials and Methods

The need for high resolution in a relatively wide operating temperature range, typical for the surface of the human body (26–34 °C), determines one of the basic requirements for the thermometer—the possibility of automatic adjustment of its parameters.

The proposed circuit innovations include:

- Implementation of a packageless (open frame) transistor in a diode connection to ensure operation speed;
- Use of the mixed analog-digital scheme of the operating point (OP) automatic adjustment just in the middle of the supply voltage, to ensure the maximum dynamic range for recording temperature oscillations when the constant level of the body surface temperature changes;
- Automatic gain control to ensure the dynamic range of temperature oscillations registration;
- Use of temperature pulses for testing and calibrating the device.

Thus, according to the medical sources, the recorder should have following capabilities:

- Conversion of fast TO (0.02–5 Hz) by semiconductor miniature silicon diode sensor to an electrical signal;
- Automatic selection of the first amplifier operating point position at the middle of the power supply voltage range;
- Slope selection of the conversion (V/°C) during temperature registration;
- Preliminary calibration of the sensitivity of the sensor (similar to the test signal of the electrocardiograph).

**Figure 1:**
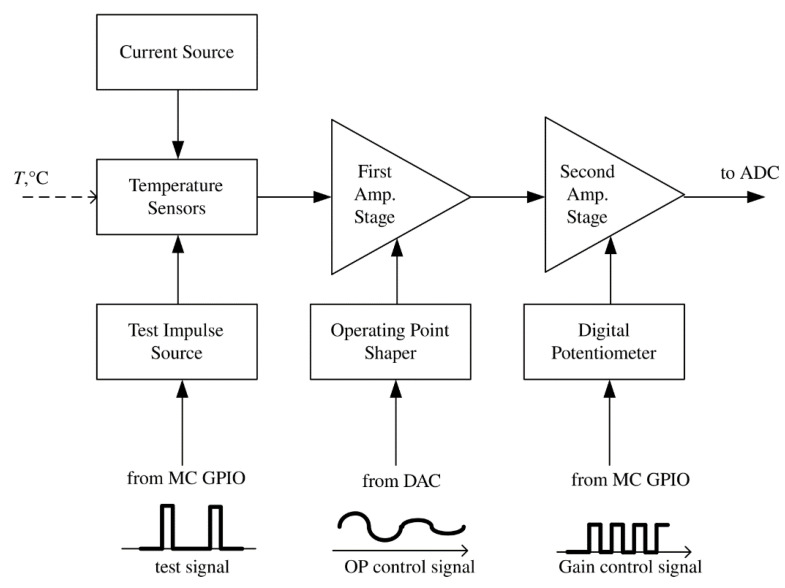
Structure of the temperature channel of the device.

According to the technical requirements, the temperature sensor was developed and constructed [10]. The proposed device is based on a combination of analog and digital solutions. Figure 1 shows the block diagram of the temperature channel of the device. The conversed signal from the output of the temperature sensor goes to the input of an analog-to-digital converter (ADC) through two amplification stages (Amp. Stage) with operating point shaping (controlled from external DAC) and gain selection (controlled with a digital potentiometer), which is carried out by the microcontroller (MC).

The main elements of the input part of the thermometer are:

- Temperature sensor—a Russian silicon chip transistor KT307 (without package, an open-frame version) in diode connection (junction base-emitter);
- Stable current source;
- The first stage of voltage amplification;
- Operating point selection block of the thermometer, i.e., the source of the compensation voltage of the initial forward diode—voltage at a given temperature;
- The second stage of voltage amplification with selectable gain;
- Source of current test impulses.

**Figure 2:**
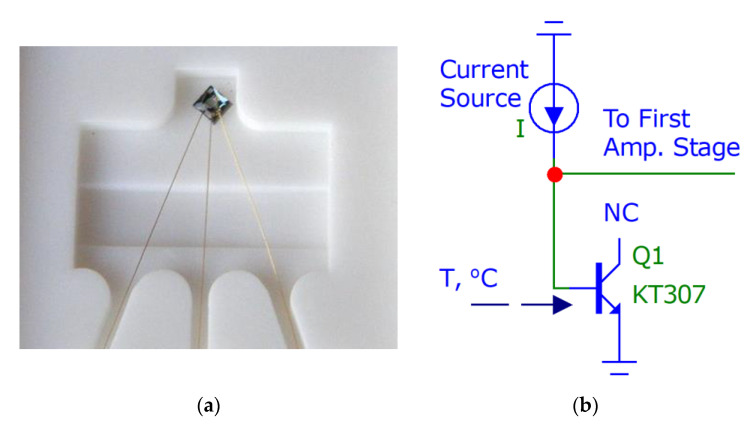
(**a**) Russian silicon chip transistor KT307 (size 0.7 × 0.7 × 0.1 mm, weight 2 mg); (**b**) connection of transistor to the current source and first amplification stage.

The temperature sensor block from Figure 1 is an open-frame silicon transistor (as can be seen in Figure 2a) with a weight of 2 mg connected in the forward direction base-emitter (the collector does not have any connection), as shown in Figure 2b.

**Figure 3:**
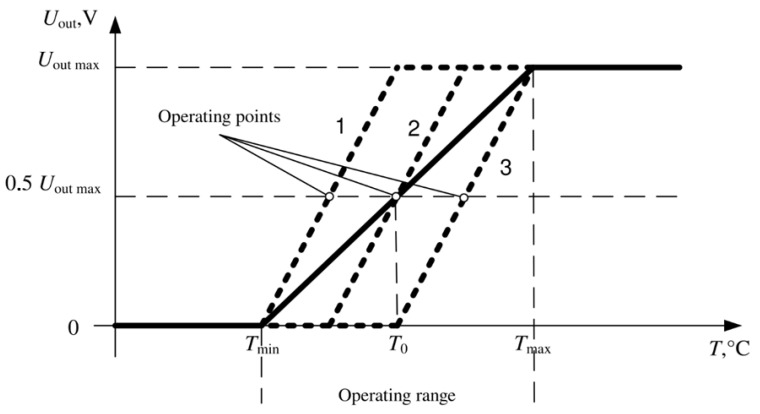
The output characteristic of the thermometer at the minimum (solid line) and the maximum gain (dashed line), curves 1, 2 and 3 are different variants of dependence in the operating range of the thermometer.

A semiconductor PN-junction in a diode and a bipolar transistor has a strong thermal dependence [16]. If the forward biased PN-junction is connected to a constant current generator with current *I*, the resulting voltage becomes a measure of the junction temperature with a rather high degree of linearity, as shown in Figure 3. The temperature-dependent voltage across the PN-junction can be expressed as Equation (1)

(1) $$V=\frac{E_{g}}{q}-\frac{2kT}{q}(\ln K-\ln I)$$

where *E_g_* is the energy band gap for silicon at absolute zero, *q* is the charge of an electron, and *K* is a temperature-independent constant. It follows from the above equation that when the junction is fed with a constant current, the voltage linearly relates to the temperature.

The maximum dynamic range of temperature recording is provided by automatically setting the position of the operating point of the first amplifying stage. The operating point before the start of the measurement is set at the middle of the supply voltage, as shown in Figure 3. The choice of the position of the output voltage (*U*_out_) for the first amplification stage is carried out by applying the compensation voltage of the initial forward diode voltage at a given temperature. This adjustment is switched on before starting the registration and maintains the indicated voltage during the entire registration period.

Due to the value limitation of the output voltage range with increasing gain, the temperature range corresponds to the linear zone.

**Figure 4:**
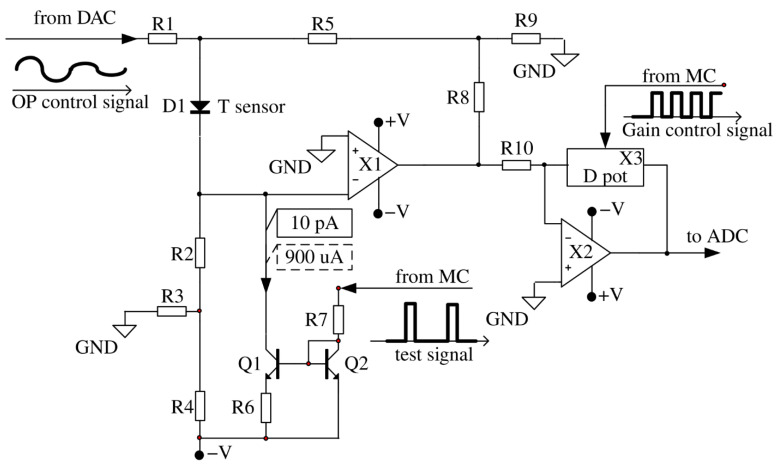
Schematic diagram of the input part of the thermometer structurally located in a separate part, located on the surface of the subject’s body. From the “from MC” inputs to the circuit, a digital signal is sent to regulate the gain of the second amplifying stage and to test the operation of the thermometer. From the “from DAC” input, an analog signal from a digital-to-analog converter (DAC) is applied to the circuit to control the position of the operating point of the first amplifier stage.

Consider the developed sensor circuit shown in Figure 4. Various versions of classical [29,30] and modern circuitry solutions [31] are known for the input cascade of the temperature sensor, however, circuit-based solutions proposed by A.V. Belov et al. in [26,27] were used in the work, which provide accurate temperature measurement using diode sensors.

The resistor R1 provides the formation of a compensation voltage for the diode direct voltage. The resistor R2 forms a constant direct current through the diode D1 (which replaces the forward direction base-emitter in the circuit to simplify the simulation). The divider R3, R4 allows to use a relatively low value of resistance R2. The voltage divider R8, R9 specifies the gain of the operational amplifier X1. In the process of temperature oscillations registration, the DC voltage supplied into the resistor R1 from the DAC does not change. The current-setting resistors (R2, R3, R4), supplied from the stabilized voltage source V, form a stable current generator, since inverting the input X1 has zero potential due to the presence of a virtual zero at its input. Thus, the resistor R1 current flows only through the resistor R5, without affecting the forward current of the diode sensor.

Transistors Q1, Q2 and resistors R6, R7 represent a controlled current generator for heating calibration impulse for the sensor. The fixed current pulses from the current generator (designed on the transistors Q1, Q2 and the resistors R6, R7), controlled by the microcontroller, are intended only for monitoring the performance of the device and calibration purposes. Due to this, the sensor slightly warms up by a fraction of a degree. During the registration of physiological signals, the sensor is not heated.

The second inverting gain stage on the operational amplifier X2 provides adjustment of the gain due to the ratio of the digital potentiometer X3 to the resistor R10.

The thermometer works as follows. The constant fixed current, which is set by the resistors R2–R4 and the supply voltage V forms a direct voltage on the diode D1 which is temperature-dependent. The temperature coefficient of the voltage for a given current is approximately −2.3 mV/°C. The compensation voltage at the diode anode is chosen equal to the diode direct voltage at a given temperature. By choosing the resistor value of R1, the output voltage is set corresponding to the minimum temperature.

At a current of 1 μA through the diode D1 and a voltage of 0.4 V, the power diffused on the PN-junction is 4 μW, which increases the p-n transition temperature due to self-heating by 0.08 °C (0.02 °C/4 μW, where 0.02 °C/μW is the value of the thermal resistance of the diode D1).

When the diode is heating, its direct voltage decreases. The decrease leads to a proportional decrease of the output voltage of the first operational amplifier X1. The compensation voltage creates a zero-output voltage by forming a forward diode voltage with a different sign. The second amplifier stage is designed to provide additional amplification, to adjust the conversion slope to a value of 2300 mV/°C. The thermometer maintains a high degree of stability of the supply voltage, since the preamplifier is very sensitive to its changes. An approximate calculation of the conversion coefficient (V/°C) range, made on the basis of the fixed gain values X1 and the adjustable gain X2, gives the following results. The diagram (Figure 4) does not show the actual values of the resistors, but they are chosen so that the maximum voltage gain in the temperature channel is 33 × 33 = 1000 (60 dB).

## 3. Results

### 3.1. Development of the Device

**Figure 5:**
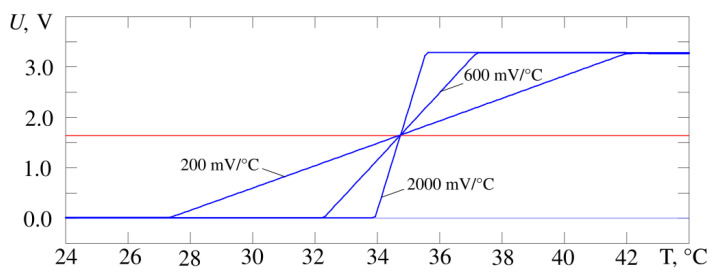
The output characteristics of the thermometer circuit (blue curves) from Figure 4, gain of cascade on Op Amp X2 is changed by a factor of 10. The operating point voltage passes at the level of the middle of dynamic range at the level of 1.64 V (red line).

**Figure 6:**
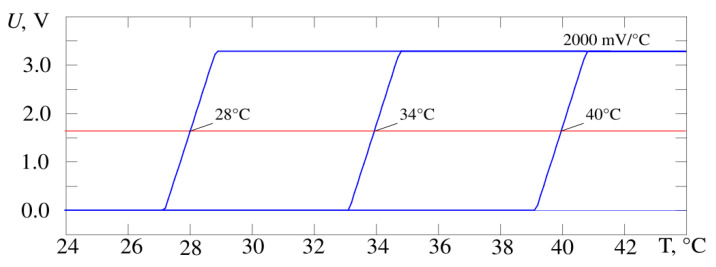
The output characteristics of the thermometer circuit (blue curves) from Figure 4, operating point temperature is changed by 12 °C. The operating point voltage passes at the level of the middle of dynamic range at the level of 1.64 V (red line).

Figure 5 shows the output characteristics of the developed thermometer circuit when the gain changes by a factor of 10 from 200 to 2000 mV/°C, Figure 6—when the operating point temperature changes by 12 °C from 28 to 40 °C. The operating point voltage has a midrange value of 1.64 V. The result is obtained by simulating the circuit in the Micro-Cap 12 software and corresponds to requirements of the proposed design in Figure 4. It is possible to adjust the gain and the operating point at the same time. The experimental data correspond to the simulation data.

According to the simulation results, the conversion coefficient values lie in the range from 200 to 2000 mV/°C (Figure 6). The slope of the characteristic U = f(T) for the whole device and its stability is determined by the selected sensor—a bipolar transistor in diode connection and the gain of the second stage on Op-Amp X2. As a result, this parameter has a fairly stable value (the scatter of values is less than 0.1% in the Microcap 12 Monte Carlo analysis). The position of the operating point is much more influenced by the instability of resistor values and amplifier parameters (variation up to 1%). In practice, the maximum value of the conversion coefficient for the developed device was 2300 ± 14 mV/°C (values of measured temperature 33.5 °C and 34.5 °C, RMS determined by results of ten measurements).

Thus, with a temperature-voltage transducer conversion factor of 2.3 mV/°C, we have a conversion coefficient value of 2300 mV/°C. Consequently, when the temperature changes by 0.01 °C, the output voltage changes by 23 mV, which, with a 16-bit ADC, will provide a code change in the 9 lower digits, which ensures a reliable fixation of temperature variations in the range from 0.01 °C. Calibration (just to calculate absolute temperature values) is performed using a specially designed thermostat.

In addition, there is a source of calibration current pulses. It used to evaluate the performance of the sensor and the slope of the temperature-voltage conversion characteristic. As described above, the calibration pulse control circuit is a current mirror formed by two identical transistors Q1 and Q2 connected to the cathode of the temperature sensor. The current flowing from the cathode of the temperature sensor D1 through the transistor Q1 is set by the resistor R7. When no voltage is applied to the input of the circuit (resistor R7), the current through Q1 is equal to 10 pA and the diode D1 works in standard mode.

The voltage impulse sent by MC increases the current which is flowing through the diode D1. This current is heating the sensor, and it could be used for evaluation of the temperature resolution at the current moment of time. Considering the circuits with an inactive and active pulse, the current through the diode D1 is 20 μA and 920 μA, and the voltage across the diode is 400 mV and 600 mV, respectively. Thus, the power released increases by ΔP which could be found by Equations (2) and (3).

P_1_ = U_1_·I_1_ = 400 × 10^−3^ V × 20 × 10^−6^ A = 8 μW, (2)

P_2_ = U_2_·I_2_ = 600 × 10^−3^ V × 980 × 10^−6^ A = 588 μW, (3)

So, ∆P = 580 μW.

The duration of the calibration impulse should be as short as possible, otherwise the ΔT from the pulse can be affected by the temperature change of the object. Therefore, the pulse duration t_imp_ is equal 100 ms.

Knowing the heating time, it is possible to find the heat Q (Equation (4)) that will be released on the sensor.

Q = P·t_imp_ = 0.5 mW×0.1 s = 0.05 mJ (4)

The heat can also be found based on Equation (5)

Q = m·c·∆T(5)

where m is the mass of the sensor, c is the heat capacity of the substance (silicon in the current case).

The value ΔT can be found from Equation (6).

(6) $$\Delta T=\frac{Q}{m\cdot c}=\frac{50\cdot 10^{-6}J}{2\cdot 10^{-6}\mathrm{kg}\cdot 714J/(\mathrm{kg}\times {}^{\circ}C)}=0.07\;{}^{\circ}C$$

**Figure 7:**
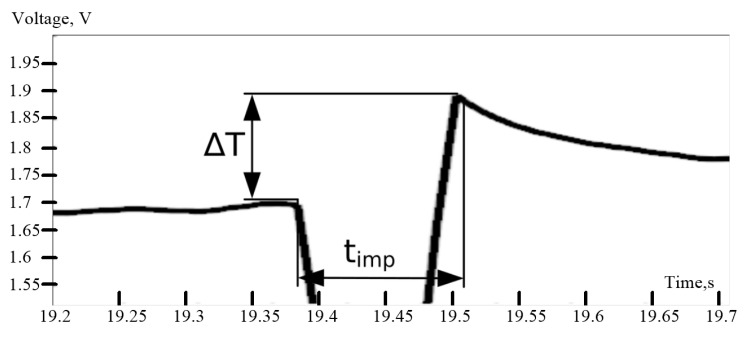
Calibration impulse of the recorder. ∆T—sensor temperature change recorded from output amplification stage (in Volts), t_imp_—duration of the impulse.

Thus, a pulse of 100 ms raises the temperature by 0.07 °C. The effect of the impulse on recoded values is shown in Figure 7. The use of a test pulse occurs in the air prior to measurements.

**Figure 8:**
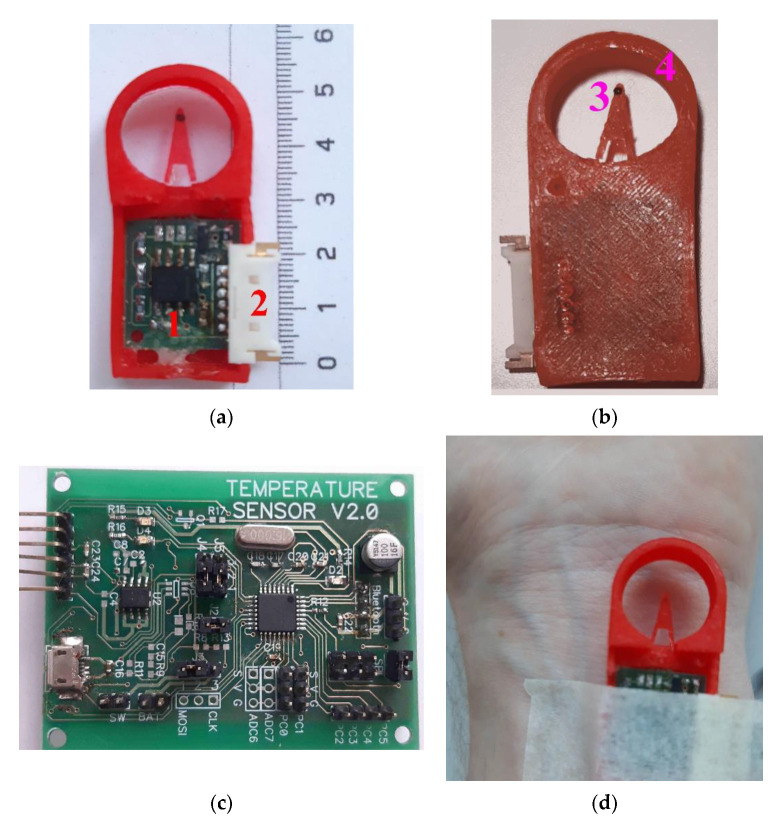
Appearance of the original construction of high-sensitive device for measurement body surface temperature oscillations: measuring part top view (**a**), 1—pre-amplifier board with control of the operating point of the first amplifying stage and the gain of the second amplifying stage, 2—connector; measuring part bottom view (**b**), 3—temperature sensor—silicon chip transistor (D1), 4—“protective ring”; processing unit top view (**c**); fixing the sensor with medical plaster in the region of the wrist joint of the right hand (**d**).

Based on the proposed schematic in Figure 4, we designed the original construction of the high-sensitive device for measurement of body surface temperature oscillations. The device itself consists of two main parts. The measuring part, shown in Figure 8a,b, where a semiconductor temperature sensor (open-frame transistor) and a preamplifier unit are installed. The main processing unit shown in Figure 8c,d, performs further analog signal processing from the sensor, analog signal conversion into digital form using an external analog-to-digital converter, data transfer to a personal computer through a Bluetooth module.

The measuring part of the thermometer is structurally remote and consists of a pre-amplifier board (Figure 8a, item 1), placed in a special case and connected through a connector (Figure 8a, item 2) and a flexible cable to the processing unit of the thermometer. The temperature sensor itself is located on the tip of the flexible tab (Figure 8b, item 3), which reduces the effect of the temperature of the pre-amplifier board on the sensor readings. To protect the sensor from external mechanical and temperature influences, a special structural element is used—the “protective ring” (Figure 8b, item 4).

The first variant of protection ring was made from Polylactic acid (PLA plastic), the most common material for 3D printing. As PLA plastic is considered as biodegradable (loses its strength over time), in the next stage of our investigation, we manufactured it from Acrylonitrile butadiene styrene (ABS) thermoplastic polymer with much more hardness and rigidity, quite suitable for making a prototype for further experiments.

### 3.2. Experiment

To verify the accurate operation of the device, two groups of experiments were carried out. During the first group of studies, the output of the thermometer was recorded when the temperature sensor was in the thermostat at a fixed temperature of 30 °C. Then, a signal was recorded when the temperature sensor was located on the surface of the skin in the region of the wrist joint of the right hand. The temperature in the thermostat and on the tested subjects were recorded at the same parameters, including the same positions of the operating point and the amplification factors of the second amplification stage.

**Figure 9:**
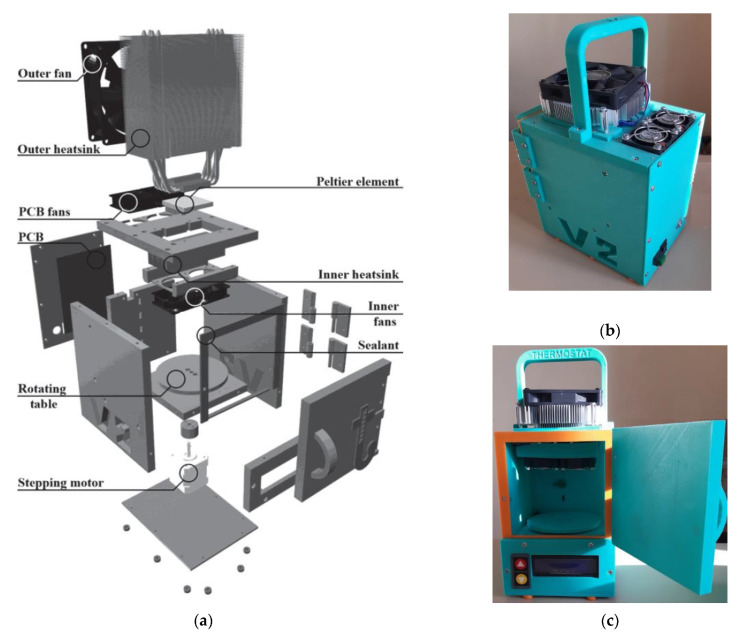
(**a**) Design of the thermostat showing internal elements; (**b**) layout of the thermostat in a 3D-printed housing, side view; (**c**) layout of the thermostat, general view.

The used thermostat is based on previous work [32]. It was improved and built specially for this experiment, as shown in Figure 9. The proposed design of the thermostat works on Peltier elements (thermoelectric converters based on the Peltier effect). The polarity of the voltage applied to the Peltier elements can be reversed with a relay, allowing not only heating, but also cooling the air in the thermostat.

The microprocessor unit controls the active cooling of the Peltier elements (which in turn are heated by cooling the air inside the thermostat) and the backlighting via a power driver. Four ADC channels are used to connect different types of sensors. An external temperature sensor is used to measure the ambient temperature. The temperature sensors used for the thermostat, type LMT-70 (Precision Analog Temperature Sensor from Texas Instruments, accuracy ±0.05 °C (typical) or ±0.13 °C (max) from 20 °C to 42 °C), have a time constant of about 1 s, which gives a quick response to any temperature changes.

The buttons on the thermostat housing are used to control the thermostat temperature. To change other parameters via wireless communication (Bluetooth channel), an application for mobile devices and personal computers has been designed. The developed device has been tested and showed parameters according to the technical requirements:

- Temperature range: +20–+40 °C;
- Accuracy of temperature measurement: 0.1 °C;
- Accuracy of temperature setting: 0.5 °C;
- Chamber volume: 1 L.

In the second group of studies, we simultaneously recorded a signal of temperature and pulse wave (with a photoplethysmogram sensor of the Biopac device, as it was described in the previous section) from young healthy subjects with a clean skin surface. The sensor was attached to the arm with an elastic band.

**Figure 10:**
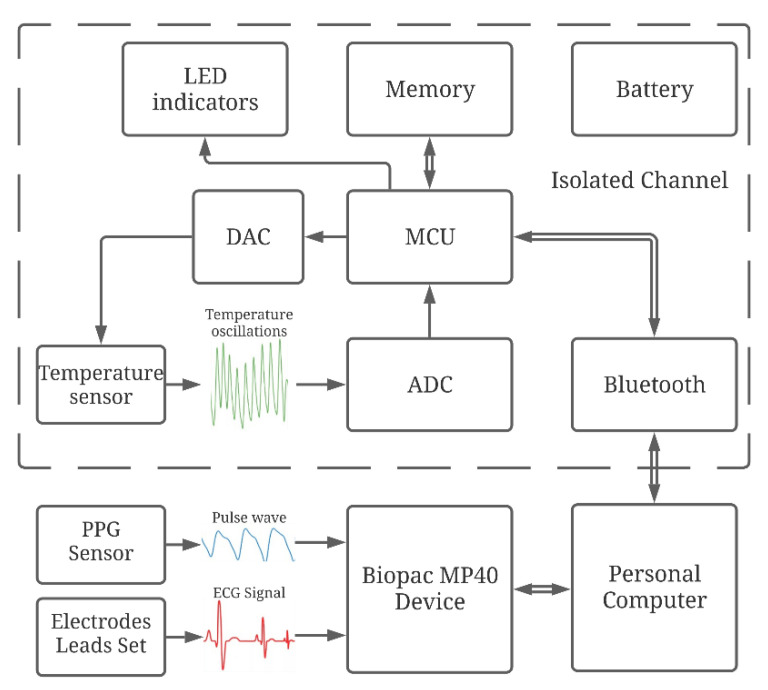
Structure diagram of laboratory setup with channels for synchronous recording temperature oscillations, PW and ECG signals.

To conduct laboratory studies, an experimental setup has been developed, as shown in Figure 10. It includes an original wireless device for recording temperature oscillations, and a data acquisition system from BIOPAC Systems, for synchronous recording of electrocardiogram (ECG) and pulse wave (PW) signals of high quality.

The remote part of the device for temperature oscillations registration is placed near the patient’s skin, for example, in the region of the wrist joint of the right hand. The skin temperature oscillations are recorded with the developed device. To calibrate and control the correct operation of the thermometer, a test signal is generated by the square-wave current generator.

Recorded input signals from sensor come from the microcontroller through Bluetooth to a computing device, for example, a personal computer (PC).

The control signals sent by the PC are transmitted to the microcontroller, to adjust the parameters of the thermometer during operation.

The microprocessor performs the following actions:

- Processing of digital output data from the ADC;
- Write the data from the ADC to the memory card;
- Transmission of recorded data from the MC to the Bluetooth module;
- Transfer of commands for controlling the parameters of the thermometer preamplifier: the test pulse source, the DAC, and the digital potentiometer.

The usage of a Bluetooth in this design additionally ensures the patient’s safety due to the presence of an electrical isolation and reduces the influence of power supply 50/60 Hz interference on the amplification stages of the thermometer. To power the device, a Li-Pol battery with a capacity of 700 mAh and a nominal voltage of 3.7 V is used. To ensure a stable 3.3 V supply voltage in the digital and analog parts, two low drop-out LDO linear voltage converters of the ADP3303 series are used. Due to the low power consumption of the device, the autonomy of its operation reaches 8 h. The level of battery discharge is carried out by voltage control. The batteries could be charged using a universal USB charger. It is possible to work from an external power source with a voltage from 3.5 to 12 V. The use of a radio channel for data transmission and an autonomous power supply significantly reduces the level of noise in the analog circuitry of the device. The digital part is described in detail in [33].

In the first version of the laboratory setup, the original device for recording ECG and pulse wave signals has been designed [34]. To record the ECG signal, a solution based on the AD8232 chip from Analog Devices has been used, with the addition a notch filter to remove the main interference (50 Hz noise [35]). As the input stage AD8232 includes an instrumental amplifier on two matched current amplifiers controlled by voltage (trans conductive operational amplifiers), as opposed to circuits using standard operational amplifiers, which allows a significant increase in the common mode rejection ratio. In addition, the input stage includes an upper-pass filter (whose cutoff frequency is set by external passive components), which allows to filter frequencies close to the constant component of the signal, which provides an initial gain of 40 dB with a drift of the signal constant component up to ±300 mV. The active ground formation block (right leg drive loop) is designed to form an inverted version of in-phase interference on the third electrode, for the purpose of additional suppression of noise.

We used two variants of circuit solution for the pulse wave registration:

- Digital, using the Texas Instruments AFE4400 Analog Front End solution, which combines in one package an input differential transimpedance amplifier, where the photodiode current is converted to voltage (with the gain adjustment set on the microcontroller side). Filtering of the signal from external light is provided by the ambient light cancellation block. The amplified signal passes through the antialiasing filter with a cut-off frequency of 500 Hz, goes to the matching buffer stage and further to the input of analog-to-digital converter (high-resolution 24 bits Sigma-Delta ADC), data in digital form go to the microcontroller through the SPI interface.
- Analog, in which the signal from the optical sensor is fed to the input differential transimpedance amplifier, to convert the photodetector current into a voltage. The converted signal is filtered (0.05 to 40 Hz) in a given spectral range, amplified (40–60 dB) and fed to the matching buffer cascade and further to the input of an analog-to-digital converter (standard ADC of successive-approximation type, 16 bits resolution).

In both cases, a Nellcor-Covidien DS100A finger clip (in photoplethysmography mode, with an infrared sensor as a light source) was used as a transducer.

**Figure 11:**
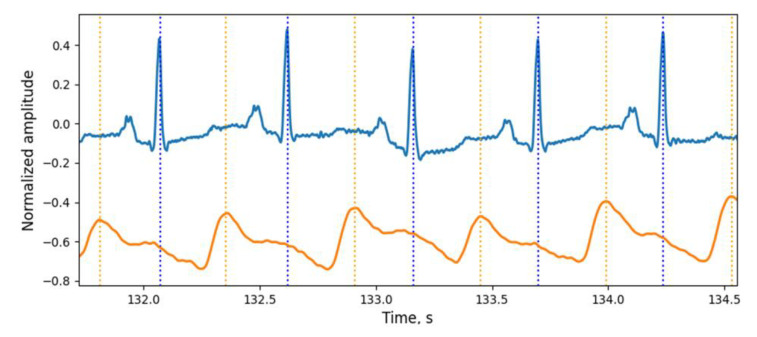
Examples of ECG and PW signals, obtained from the certified Biopac MP36 data logger (medical-grade quality of signals).

For the new stage of investigation, the simultaneous recording of ECG and PPG signals was performed using a Biopac polysomnographic system (MP36 Data Acquisition Unit, because it is certified as a medical device) with a sampling rate of 500 Hz for each channel. The ECG signal was recorded from the hands of the subject using the electrode lead set SS2LB and disposable electrodes. The PPG signal was recorded from the right index finger using an SS4LA optical sensor working on reflection. Each signal was pre-filtered with the MP36 data logger and then analyzed on a personal computer. Examples of the signals received are shown in Figure 11.

Data on the maximum of the pulse wave and R-wave of the ECG signal serve for two goals. First, they make it possible to detect and verify rapid temperature oscillations (an example of such a verification is shown below). The second, temporal relationship between the signals of temperature oscillations, pulse wave and ECG signal have an important physiological significance. The phase relationship between these signals allows us to estimate the propagation time of the pulse wave and temperature time constant of the surface tissues of the body.

The change in the parameters of the temperature oscillations corresponding to the transient processes during occlusive [36] or orthostatic samples [37], can serve as an indicator of various pathological states of a microcirculatory channel. These changes allow us to calculate the reserve of capillary blood flow characterizing the adaptive reserves of the microcirculation system. The temperature constant of time characterizes the reactivity of the blood vessels microcirculatory bed and is determined by the number of vascular blocks and the degree of ischemia of the examined body area.

## 4. Discussion

**Figure 12:**
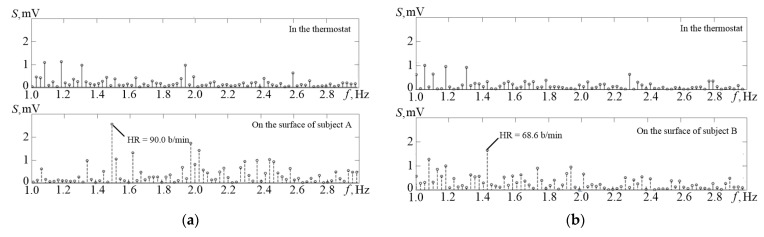
Comparison of the amplitude spectrum of the TO signals obtained when the temperature sensor is placed into the thermostat (the upper parts of graphs (**a**,**b**)) and when placed on the surface of the body of test A and test B (the lower parts of graphs (**a**,**b**)). The lower graphs clearly distinguish the frequency components corresponding to the average pulse frequencies of the subjects, 90.0 and 68.6 bpm.

Figure 12 shows the amplitude spectrum (S on the graph) of the temperature channel signals obtained in the thermostat (upper graph) and the amplitude spectrum of the temperature signal recorded from the surface of the patient’s skin. It can be identified on the lower graph in Figure 12a that it is possible to extract an obvious spectral component of the pulse at 1.08 Hz, which corresponds to the heart rate of 65 beats per minute.

**Figure 13:**
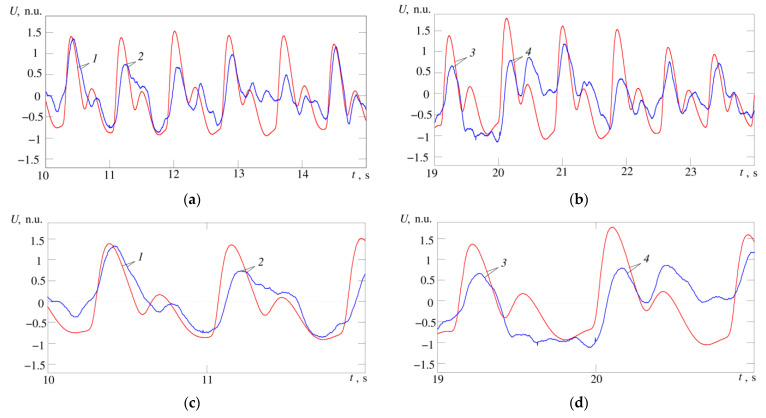
Fragments of the recorded temperature signal (blue curve) and pulse wave (red curve); (**a**) records for subject A; (**b**) records for subject B; (**c**,**d**) the same waveforms on a larger scale for subject A and B, respectively; 1–4 are the maximum cusps of the compared cardiac cycles by the temperature and pulse wave signals. The correlation is 0.65 (**a**) and 0.54 (**b**), (*p* < 0.05). Signal amplitude values are normalized, n.u.—normalized units.

In Figure 13a,b maximum cardiocycle waveforms for temperature and pulse wave signals are shown for comparison; in Figure 13c,d, the same waveforms are shown in a larger scale. 1—signals of temperature and pulse wave at the limits of the cardiocycle practically coincide, in the temperature signal we can easily distinguish phases corresponding to anacrota, catacrota, incisura and dicrotic wave. 2—temperature and pulse wave signals are similar, but the phase corresponding to the incisura cannot be clearly distinguished in the temperature signal. 3—temperature and pulse wave signals coincide in the anacrota and catacrota phases, but the temperature signal lacks phases corresponding to the incisura and dicrotic wave. 4—temperature and pulse wave signals are close in shape and all four phases can be easily distinguished in the temperature signal, but in the phase corresponding to the dicrotic wave, the temperature signal has values at the level of the maximum anacrota.

The slow wave changes in the pulse amplitude in Figure 13b may indicate differences in capillary functioning (more or less vasomotion). The changes in amplitude over time scales of multiple heartbeats may also be related to vasomotion, but this assumption requires additional experiments.

As in the study of electrocardiographic or pulse wave signals, the results of recording temperature oscillations strongly depend on the location of the temperature sensors. A thermal signal has a large amplitude if the temperature sensor is located near large arteries, for example, a carotid. However, temperature oscillations can be recorded at various sites of the skin surface of the body. Thus, a system designed to study physiological processes of blood macro- and microcirculation using recordings of surface temperature oscillations has been developed. The most important condition for obtaining data on blood circulation in the developed system was the presence of synchronously obtained data at three sites of blood flow formation:

1. In the source of mechanical power of blood flow—in the heart, this is the ECG signal;
2. In peripheral vessels, this is the pulse wave of blood filling plethysmogram;
3. In peripheral capillary channel, this is the signal of surface temperature oscillations.

The first component is connected with the cardiac performance and characterized by HRV parameters, the second with the state of vessels (total peripheral resistance and total elasticity of vessels), the third with the action of fluctuations in the blood flow. The phase relations of the indicated signals and their frequency characteristics reflecting the oscillatory nature of blood flow formation processes are also of importance.

As mentioned above, the variability of microcirculation characterizes the level of tissue vitality, and its rhythmic oscillations in different frequency ranges provide information about the relationships in the action of different regulatory mechanisms. That is why the joint analysis of the frequency components of HRV signals, plethysmography and surface temperature oscillations seems rather promising.

## 5. Conclusions

As is known from numerous studies, physiological activity causes changes in the body surface temperature in a wide amplitude and frequency range, associated with heartbeat pulsations, which have a very small amplitude and maximum frequency of temperature fluctuations. Their registration requires devices with extremely high resolution and speed, which are not available on the market.

To solve this problem, an original circuit solution was proposed, where the required thermal time constant is achieved by using a semiconductor diode sensor in an open frame design. The need for high resolution in a relatively wide range of operating temperatures determined one of the main features of our solution—the possibility of automatic adjustment of parameters, such as the gain and position of the operating point.

Thus, with a semiconductor diode conversion coefficient of temperature to voltage of 2.3 mV/c, at the output of the developed thermometer, we have a conversion coefficient value of 2300 mV/°C. Consequently, when the temperature changes by 0.01 °C, the output voltage changes by 23 mV; thus, combined with at least 16-bit ADC, it provides a reliable fixation of temperature changes in a wide range from 0.01 °C. An original design of a small volume thermostat on Peltier elements has been developed for calibration and calculation of absolute temperature values.

To carry out laboratory research of the proposed solution, an experimental setup including an original wireless device for recording temperature variations and a recorder of physiological signals made by Biopac for synchronous recording of electrocardiogram and pulse signals has been designed. The results obtained (comparison of temperature oscillations and pulse wave signal recorded from the surface of the human body) has shown that the design approach allows investigating blood microcirculation.

Nevertheless, some questions remained unresolved: first, it concerns a reliable and user-friendly mounting of the sensor to the body surface, since the structure of the open frame diode is extremely fragile and should be protected from external influence.

In the development of the proposed idea, the observation of a subject’s condition using a three-channel system (ECG, Pulse Wave and fast temperature oscillations) during various physiological loads provides an opportunity to study a complex of physiological reactions and mechanisms of cardiovascular, central and vegetative nervous systems. Further works on the presented project have the ultimate goal to develop scientifically grounded recommendations for their practical application as a unique method of diagnostics of the human condition and estimation of functional capabilities of the human organism. The obtained results will serve as a basis for the development new methods of functional diagnostics and professional selection.

As the main result of this work, the biomedical thermometer was built, tested and patented [13]. The developed device has technical characteristics (presented previously in [10]):

- Temperature range: from +20 °C to + 45 °C;
- Temperature resolution: 0.01 °C;
- Thermal time constant: 50 ms;
- Thermal resistance: 3 °C/mW;
- Sampling frequency: 0.01–1000 Hz;
- The value of test pulse: 1 °C.

## Figures and Tables

**Table 1:** Comparison of the frequency ranges of oscillations identified in the analysis of heart rate—HRV, laser Doppler flowmetry (LDF) signals, temperature oscillations [6,7].

Oscillatory Processes in Systemic Hemodynamics (HRV)		Oscillatory Processes in Blood Microcirculation (LDF and Temperature Oscillations)	
Band Designation	Frequency Ranges, Hz	Band Designation	Frequency Ranges, Hz
		Cardiac (pulse)	0.5–2
HF	0.15–0,4	Breath	0.14–0.5
LF	0.04–0.15	Myogenic	0.05–0.14
VLF	0.003–0.04	Neurogenic	0.02–0.05
		Endothelial	0.001–0.02
UVLF	Less than 0.003	Endothelial 2	Less than 0.001

## Data Availability

Not applicable.
